# A Point Mutation in *cycA* Partially Contributes to the D-cycloserine Resistance Trait of *Mycobacterium bovis* BCG Vaccine Strains

**DOI:** 10.1371/journal.pone.0043467

**Published:** 2012-08-17

**Authors:** Jeffrey M. Chen, Swapna Uplekar, Stephen V. Gordon, Stewart T. Cole

**Affiliations:** 1 École Polytechnique Fédérale de Lausanne, Global Health Institute, Lausanne, Switzerland; 2 Schools of Veterinary Medicine, Biomolecular and Biomedical Science, Medicine and Medical Science, and the Conway Institute, University College Dublin, Dublin, Ireland; University of Padova, Italy

## Abstract

In mycobacteria, CycA a D-serine, L- and D-alanine, and glycine transporter also functions in the uptake of D-cycloserine, an important second-line anti-tubercular drug. A single nucleotide polymorphism identified in the *cycA* gene of BCG was hypothesized to contribute to the increased resistance of *Mycobacterium bovis* bacillus Calmette-Guérin (BCG) to D-cycloserine compared to wild-type *Mycobacterium tuberculosis* or *Mycobacterium bovis*. Working along these lines, a merodiploid strain of BCG expressing *Mycobacterium tuberculosis* CycA was generated and found to exhibit increased susceptibility to D-cycloserine albeit not to the same extent as wild-type *Mycobacterium tuberculosis* or *Mycobacterium bovis*. In addition, recombinant *Mycobacterium smegmatis* strains expressing either *Mycobacterium tuberculosis* or *Mycobacterium bovis* CycA but not BCG CycA were rendered more susceptible to D-cycloserine. These findings support the notion that CycA-mediated uptake in BCG is impaired as a result of a single nucleotide polymorphism; however, the partial contribution of this impairment to D-cycloserine resistance suggests the involvement of additional genetic lesions in this phenotype.

## Introduction

*Mycobacterium bovis* bacillus Calmette-Guérin (BCG), is a live vaccine originally derived from a virulent isolate of *Mycobacterium bovis*, and has been used to immunize more than three billion people against tuberculosis [1]. Despite widespread use, the protective efficacy imparted by BCG wanes significantly with time, and the molecular mechanism for this is poorly understood [1], [2]. Additionally, the mechanisms underlying the derivation of attenuated BCG from virulent *M. bovis* also remain incompletely understood [1], [2]. To further complicate matters, 13 strains of BCG with documented differences in attenuation and protective efficacy currently exist, and several of these are in use in various parts of the world [3], [4]. Clearly, the basic biology of BCG is far from being fully understood. The availability and simultaneous analyses of complete genome sequences of several *M. bovis* and BCG strains however have generated fresh insights into the biology of BCG [1], [4]–[6]. Indeed, the demonstration that loss of the RD1 locus by BCG was a key event leading to its attenuation, originated from such comparative genome studies [7].

D-cycloserine (DCS), a cyclic analog of D-alanine, is an important second-line antibiotic used to treat multi-drug- and extensively drug-resistant *Mycobacterium tuberculosis* infections [8], [9]. Compared to wild-type *M. tuberculosis* and *M. bovis*, BCG has always been found to be more resistant to DCS [6], [10]. Although the molecular basis for this phenotype is unknown, this feature is used to differentiate the vaccine strain from *M. bovis* and *M. tuberculosis* strains [10]. *In vitro*, DCS inhibits *M. tuberculosis* alanine racemase (Alr) which converts L-alanine to D-alanine, and D-alanine:D-alanine ligase (Ddl) which synthesizes D-alanine pentapeptides [8], [11], [12]. Both enzymes are required for the synthesis of peptidoglycan in the cell wall of mycobacteria [8], [11], [12]. Overproduction of Alr and Ddl in BCG and *Mycobacterium smegmatis* confers increased DCS resistance [13], [14], while their genetic inactivation in *M. smegmatis* increases susceptibility to the drug [15], [16]. The resistance determinants and the cellular targets of DCS in *M. tuberculosis* and *M. bovis* are presumed to be Alr and Ddl, both of which are essential in *M. tuberculosis* [17]. However, the nucleotide sequences and expression patterns of *alr* and *ddl* in *M. bovis* and BCG appear to be identical and as such, might not contribute to the DCS resistance of the vaccine strain [1], [6].

A non-synonymous single nucleotide polymorphism (nsSNP) in the *cycA* gene of all BCG strains was recently identified and predicted to result in a glycine 122 to serine substitution (G122S) in the transporter [6]. CycA is a bacterial D-serine/L- and D-alanine/glycine/D-cycloserine: proton symporter, comprising 12 helical trans-membrane domains, of the amino acid transporter (AAT) family (TCID: 2.A.3.1.7) [18]. In the early 1970’s, David isolated and characterised two low-level and equally DCS-resistant forms of *M. tuberculosis* mutants - one that was also D-serine, L- and D-alanine, and glycine uptake defective, and the other uptake competent [19]. Point mutations resulting in transitions and transversions, as well as small deletions and duplications within the *cycA* gene of *Escherichia coli*, have been found to confer resistance to DCS [20]. Inspired by these reports, it was hypothesized that the G122S change functionally impairs BCG CycA and contributes to the vaccine strain’s characteristic resistance to DCS [6].

A bioinformatics analysis shows that the G122S change in BCG CycA lies in a presumably critical extracellular loop between the conserved third and fourth helical trans-membrane domains. Taking a genetic approach we show that BCG CycA is indeed impaired for DCS uptake, however the G122S change only partially contributes to DCS resistance in BCG, thus implicating additional mutations in this phenotype.

## Results

### *In silico* Analyses and Helical Trans-membrane Domain Modeling Predicts a Functionally Important Extracellular Loop in CycA

**Figure 1:**
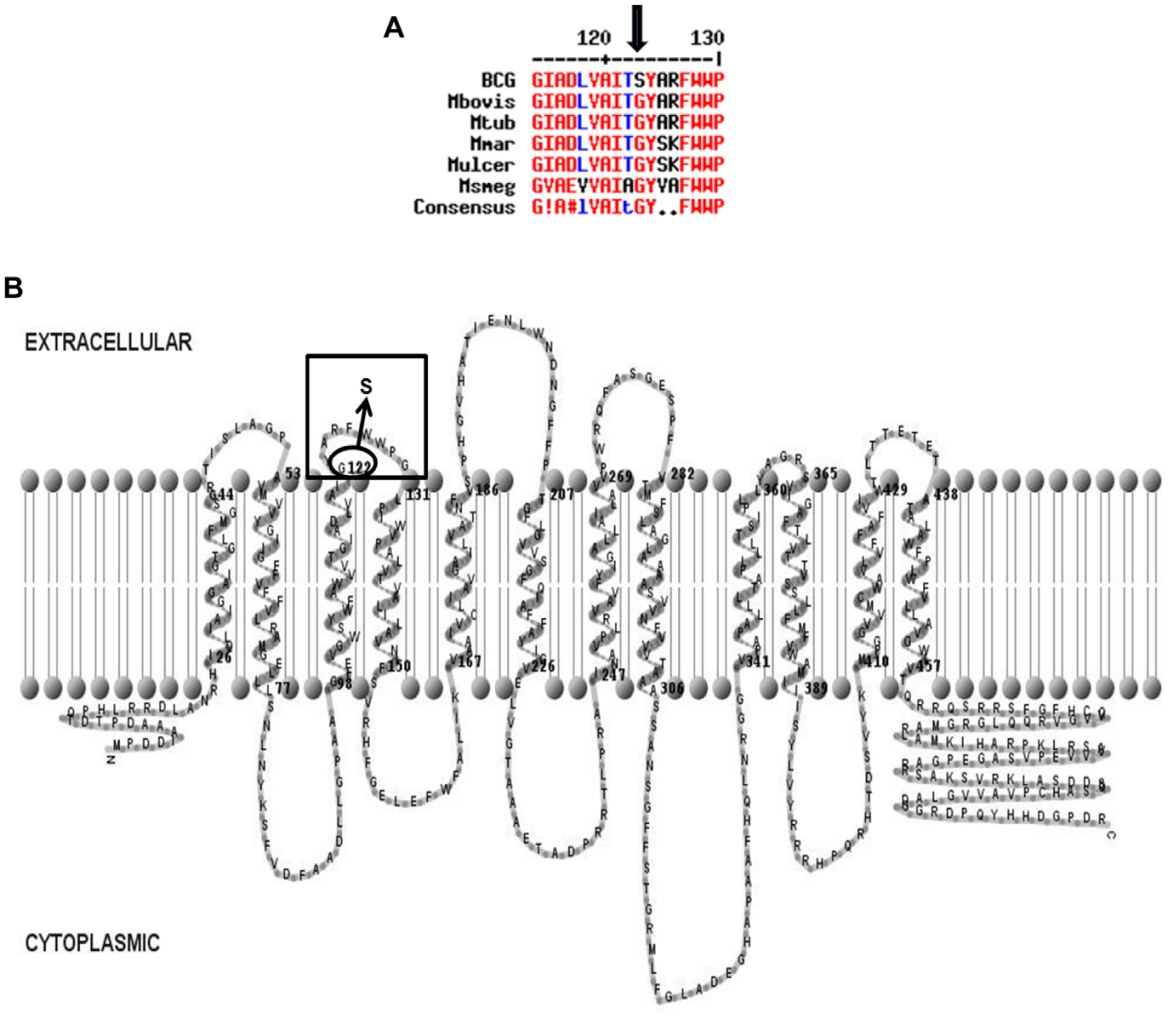
Analyses of CycA amino acid sequences. (**A**) Partial CycA amino acid sequence alignment of various mycobacteria (the conserved G122 residue is indicated by the arrow). (**B**) 2-dimensional topological representation of *M. bovis* CycA with the G122S mutation found in all BCG (circled) in the extracellular loop (boxed) between the 3^rd^ and 4^th^ trans-membrane helices from the amino (N) terminus.

Comparison of CycA orthologues from six different mycobacteria revealed 72–99% identity at the amino acid sequence level. Multi-species protein sequence alignment shows that CycA from all mycobacterial strains, except BCG possess a conserved glycine residue in position 122 (Figure 1A). Helical trans-membrane (HTM) domain modeling of the *M. bovis* AF 2122/97 CycA based on HMMTOP prediction [21], shows the transporter to be a cell membrane protein with 12 HTM domains (Figure 1B). The G122S change in BCG CycA is predicted to lie in an extracellular loop between the third and fourth trans-membrane helices (Figure 1B). Similar modeling of the HTM domains of *M. tuberculosis* H37Rv, *M. marinum* M and *M. smegmatis* mc^2^155 CycA also place the conserved glycine residue at the beginning of the extracellular loop between the third and fourth trans-membrane helices. Single point mutations in the *cycA* gene have been identified in DCS-resistant *E. coli* mutants [20]. Therefore *E. coli* CycA was also modeled and DCS-resistance-associated single point mutations were mapped to the model. Like the mycobacterial CycA proteins, *E. coli* CycA was predicted to form a membrane protein with 12 HTM domains. Of twenty previously identified point mutations, we were able to place a threonine 114 to proline substitution (from an adenine to cytosine transversion) [20], in the extracellular loop between the third and fourth trans-membrane helices of *E. coli* CycA (Figure S1).

Our *in silico* analyses suggest that the extracellular loop between the third and fourth trans-membrane helices of CycA may be sensitive to modifications and is functionally important for transport activity.

### Merodiploid Expression of *M. tuberculosis* CycA in BCG Partially Restores Susceptibility to D-cycloserine

**Figure 2:**
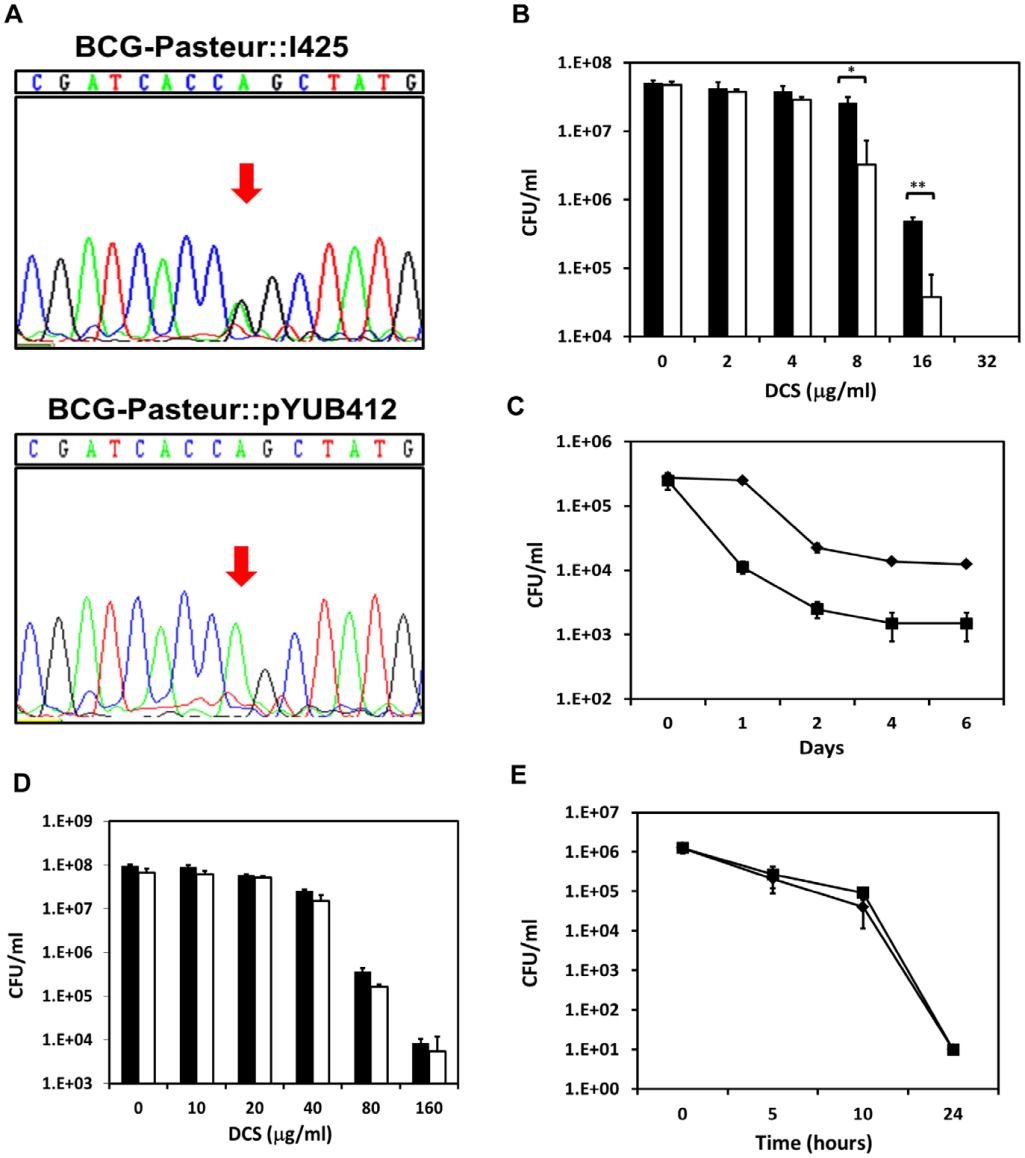
Characterization of *cycA* merodiploid BCG and *M. smegmatis* strains. (**A**) Sequence of the SNP containing region in *cycA* from BCG-Pasteur::I425 and BCG-Pasteur::pYUB412 clones from the 5′ end. (**B**) CFUs of BCG-Pasteur::I425 (white bars) and BCG-Pasteur::pYUB412 (black bars) 4 days after treatment with indicated concentrations of DCS. (**C**) CFUs of BCG::I425 (solid squares) and BCG::pYUB412 (solid diamonds) obtained over time upon exposure to 32 µg/mL DCS. (**D**) CFUs of *M. smegmatis*::I425 (white bars) and *M. smegmatis*::pYUB412 (black bars) 24 hours after treatment with indicated concentrations of DCS. (**E**) CFUs of *M. smegmatis*::I425 (solid squares) and *M. smegmatis*::pYUB412 (solid diamonds) obtained over time upon exposure to 200 µg/mL DCS. Data points and error bars are means ± standard deviations. Statistically significant differences are denoted by asterisks (*, P<0.05; **, P<0.01). Representative of 3 independent experiments performed in duplicate.

A merodiploid strain of *M. bovis* BCG-Pasteur was generated by transformation with an integrating cosmid (I425) containing the *M. tuberculosis cycA* allele. Sequencing from the 5′-end of the *cycA* PCR product amplified from the genomic DNA of a Pasteur::I425 knock-in clone showed both GGC (encoding Gly) and AGC (encoding Ser) sequences, indicating the successful integration of the cosmid into the chromosome (Figure 2A). In contrast, a Pasteur::pYUB412 control showed only the AGC (encoding Ser) sequence (Figure 2A). 3′-end sequencing of *cycA* PCR products from both clones confirmed these findings.

The susceptibility of the Pasteur::I425 knock-in strain to DCS was assessed and compared to that of the Pasteur::pYUB412 control strain. In 7H9 broth cultures containing increasing concentrations of DCS (0, 2, 4, 8, 16 and 32 µg/mL), the Pasteur::pYUB412 strain showed complete growth inhibition at >32 µg/mL of the antibiotic, which is consistent with published observations [10], [13], [22]. The Pasteur::I425 strain however, appeared to be more susceptible to DCS, exhibiting significant growth inhibition at 16 and 8 µg/mL DCS compared to the control strain – the colony forming units of the Pasteur::I425 strain recovered was almost 1-log less at these concentrations of DCS than that of the control strain (Figure 2B). The difference in susceptibility to DCS between the two strains was even more pronounced in experiments measuring growth inhibition over time upon exposure to 32 µg/mL of the antibiotic. Pasteur::I425 showed more rapid inhibition of growth by DCS compared to the Pasteur::pYUB412 strain as the colony forming units of the knock-in strain recovered at all time- points post-exposure was consistently 1-log lower than the control strain (Figure 2C). However, we did not observe in the Pasteur::I425 strain, the restoration of wild-type *M. tuberculosis* or *M. bovis* levels of DCS susceptibility, which is reportedly between 4 and 5 µg/mL of DCS [10], [19].

We next wanted to verify that the higher susceptibility of the Pasteur::I425 strain to DCS was not due to the integration of an extra copy of the *cycA* gene. Although *M. smegmatis* mc^2^155 has a higher MIC for DCS [13], [14] compared to members of the *M. tuberculosis* complex, we transformed this strain with I425 and pYUB412 to test if we could obtain a similar fold increase in susceptibility to DCS as seen with BCG. Using *M. tuberculosis cycA*-specific primers we confirmed the successful integration of the cosmid prior to testing with DCS (data not shown). Growth of wild-type *M. smegmatis* mc^2^155 is inhibited by DCS at ≥75 µg/mL [13], [14], and consistent with this, the *M. smegmatis*::pYUB412 strain exhibited complete growth inhibition at ≥80 µg/mL DCS (Figure 2D). The growth of the *M. smegmatis*::I425 knock-in strain was inhibited by DCS to the same extent as the *M. smegmatis*::pYUB412 control strain (Figure 2D). Likewise, time-course experiments measuring growth inhibition by DCS also did not show significant differences between the two strains (Figure 2E). This shows that having one extra copy of *M. tuberculosis cycA* does not appreciably increase DCS susceptibility in *M. smegmatis*. All of these observations taken together indicate that the increased DCS susceptibility of Pasteur::I425 results from the production of a functional CycA in a genetic background expressing either a non-functional or impaired CycA.

### *M. smegmatis* Overexpressing BCG CycA does not become More Susceptible to DCS

Heterologous expression of *M. tuberculosis* proteins involved in efflux and drug resistance in *M. smegmatis* has been an effective approach to study their function [23]–[25]. We hypothesized that if *M. bovis* BCG CycA is defective, then *M. smegmatis* strains overexpressing functional *M. tuberculosis* and *M. bovis* CycA but not BCG CycA, would become more susceptible to DCS-mediated growth inhibition. Working along these lines, *M. tuberculosis*, *M. bovis* and BCG *cycA* alleles and their promoter regions were individually cloned into the pMD31 plasmid for multi-copy ectopic expression in *M. smegmatis* mc^2^155. Colony PCR was used to discriminate *bona fide* transformants harbouring plasmids containing *M. tuberculosis*, *M. bovis* or BCG *cycA* alleles from those harbouring the empty pMD31 vector alone (data not shown).

**Figure 3:**
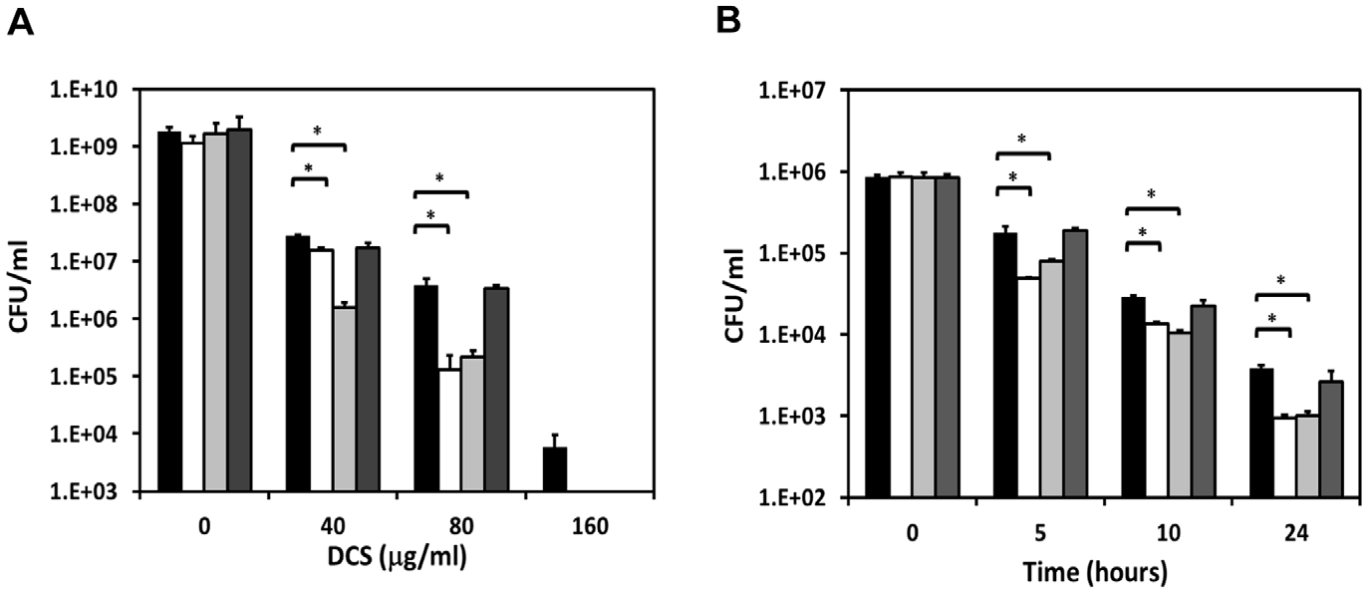
Susceptibilities of *M. smegmatis* strains overexpressing *cycA* alleles to DCS. (**A**) CFUs of recombinant *M. smegmatis* strains after 24 hours of treatment with indicated concentrations of DCS. (**C**) CFUs of recombinant *M. smegmatis* strains obtained over time upon exposure 200 µg/mL DCS. *M. smegmatis* harbouring pMD31 (black bars), pMDcycA_Mtb_ (white bars), pMDcycA_Mbov_ (light-gray bars) and pMDcycA_BCG_ (dark-gray bars). Data points and error bars are means ± standard deviations. Statistically significant differences are denoted by asterisks (*, P<0.05). Representative of three independent experiments performed in duplicate.

The DCS sensitivities of these recombinant *M. smegmatis* strains were then tested in 7H9 broth containing increasing concentrations of DCS (0, 40, 80 or 160 µg/mL). *M. smegmatis* strains producing *M. tuberculosis* and *M. bovis* CycA exhibited significantly increased susceptibility to DCS-mediated growth inhibition compared to the vector-only control strain (Figure 3A). In contrast, the susceptibility of *M. smegmatis* producing BCG CycA was similar to that of the vector-only control strain (Figure 3A). Consistent with this observation, in the presence of 200 µg/mL DCS, only *M. smegmatis* strains producing *M. tuberculosis* and *M. bovis* CycA but not BCG CycA exhibited significantly faster growth inhibition compared to the vector-only control (Figure 3B). These results indicate that raising the gene-dosage of *cycA* readily increases DCS susceptibility in *M. smegmatis*. More importantly it shows that BCG CycA is defective because even in the *M. smegmatis* genetic background, raising the gene-dosage of BCG *cycA* does not affect its susceptibility to DCS.

### Extra Copies of *cycA* Alleles in Mycobacteria do not Confer an in vitro Growth Advantage

Since CycA is a D-serine, L- and D-alanine, and glycine transporter, we wanted to verify that the differences in DCS susceptibilities observed were not due to changes in the growth rates of the strains tested. Accordingly growth measurements were made for all of the strains in 7H9 broth. No difference in growth between the merodiploid Pasteur::I425 and the Pasteur::pYUB412 control strain were found (data not shown). Similarly no growth differences between *M. smegmatis*::I425 and *M. smegmatis*::pYUB412 were observed (data not shown). Consistent with the above results, *M. smegmatis* strains producing *M. tuberculosis*, *M. bovis* and BCG CycA, also grew at rates similar to the control strain in 7H9 media (data not shown). These findings indicate that expression of extra copies of *cycA* do not confer a growth advantage to the host strain in the culture media used to test for susceptibility to DCS.

## Discussion

We employed bioinformatics as well as two complementary genetic approaches to investigate the involvement of the nsSNP in BCG *cycA* in DCS resistance. Evidence is provided that suggests this nsSNP causes a defect in the BCG CycA transporter but only partially contributes to DCS resistance in the vaccine strain.

Trans-membrane helical domain modeling places the BCG G122S mutation in an extracellular loop between the conserved third and fourth helical trans-membrane domains of CycA. This finding hints at possible substrate recognition or binding defects at the structural level in BCG CycA and warrants further study. Consistent with the notion that BCG CycA is defective for DCS uptake, heterologous overexpression of CycA from *M. tuberculosis* and *M. bovis* but not from BCG in *M. smegmatis* results in increased susceptibility to DCS. This effect of increased *cycA* gene-dosage on DCS susceptibility in *M. smegmatis* raises the question of whether a decrease in CycA expression in BCG might result in increased resistance. However, the identical expression patterns of *cycA* in *M. bovis* and BCG rule out this possibility [1], [6]. More importantly, the G122S mutation in BCG CycA appears to be a minor contributor as we were unable to restore *M. tuberculosis* and *M. bovis* levels of DCS susceptibility to the merodiploid Pasteur::I425 strain. This is not surprising as the isolation of two equally DCS-resistant mutants of *M. tuberculosis*, one of which is transport defective and the other transport competent, has been reported [19]. This strongly suggests there are other genetic lesions besides the nsSNP in *cycA* that also contribute to DCS resistance in BCG. Indeed an *M. smegmatis* isolate, resistant to both DCS and vancomycin was found to have no mutations in the *alr*, *ddl* or *cycA* genes [26]. Only multi-copy transformation of a gene encoding a putative penicillin-binding protein (Pbp4) homolog restored DCS and vancomycin susceptibility to this strain [26]. Our analyses of *pbp* genes in BCG however did not reveal any nsSNPs that might explain the BCG phenotype. Moreover, a recent metabolomic study of *M. smegmatis* revealed that there may potentially be alternative routes to D-alanine synthesis in mycobacteria that could circumvent DCS inactivation of Alr and Ddl [12]. This notion is supported by the observation that *alr* mutants of *M. smegmatis* are not complete D-alanine auxotrophs [12], [16]. As such, genetic mutations in these alternate alanine metabolic pathways could potentially augment CycA-mediated DCS resistance in BCG.

BCG underwent *in vitro* evolution during its initial derivation from a virulent *M. bovis* progenitor strain as well as during its subsequent global dissemination [1], [2]. A consequence of this evolution was the acquisition of mutations causing defects in L-serine and L-alanine catabolism, dysregulated nitrogen metabolic pathways and *in vitro* growth defects [27]. CycA is the sole transporter for D-serine, L- and D-alanine, and glycine in mycobacteria, and their uptake by the transporter is induced by L-alanine [19]. Although in this study BCG and *M. smegmatis* strains bearing extra *cycA* alleles and their control strains grew similarly in 7H9 broth, growth differences may yet appear in L-serine and/or L-alanine-replete conditions not found in 7H9 broth. A link between defective L-serine and L-alanine catabolism and the acquisition of the CycA G122S mutation impairing D-serine, L- and D-alanine and DCS uptake is intriguing and merits additional investigation as this will add to our understanding of BCG nitrogen metabolism.

In conclusion, this study reveals the involvement of CycA in the resistance of BCG to DCS and provides fresh insights into BCG biology as well as clues for the improvement of DCS as an anti-mycobacterial agent.

## Materials and Methods

### Reagents

Restriction and DNA modifying enzymes were purchased from New England Biolabs (Ipswich, MA, USA). High fidelity *Pfu* polymerase for PCR was purchased from Promega (Madison, WI, USA). Custom oligonucleotides were synthesized by Microsynth (Balgach, Switzerland). Middlebrook 7H9, 7H11 media, albumin-dextrose-catalase (ADC) and oleic acid-albumin-dextrose-catalase (OADC) were purchased from Becton-Dickinson (Franklin Lakes, NJ, USA). All other chemicals and reagents were purchased from Sigma-Aldrich (St. Louis, MO, USA).

### Bacterial Strains and Growth Conditions

*M. tuberculosis* H37Rv, *M. bovis* BCG Pasteur 1173P2 and *M. smegmatis* mc^2^155 were routinely cultured in 7H9 broth supplemented with ADC and 0.05% Tween-80 or on 7H11 agar plates supplemented with OADC. For the selection of relevant mycobacterial clones, hygromycin at 50 µg/mL and kanamycin at 25 µg/mL were used.

### Genetic Manipulations and Genotyping

An integrating cosmid (I425) [28], bearing the *M. tuberculosis cycA* allele (*rv1704c*) was transformed into *M. bovis* BCG-Pasteur 1173P2 and *M. smegmatis* mc^2^155 following standard electroporation procedures [29]. pYUB412, the cosmid backbone, was electroporated to obtain parental control strains. Genomic DNA was extracted from several *M. bovis* BCG colonies obtained after selection on 7H11 agar plates containing hygromycin. Positive BCG-Pasteur::I425 clones were verified by PCR amplification of the genomic DNA using *cycA*-specific internal primers (*cycA*-int-Forward: agctgctgctgtcgaacctg and *cycA*-int-Reverse: gttgggaagaacccgttgtc) and sequencing of the PCR products. *M. smegmatis*::I425 clones were obtained and identified by PCR amplification using *M. tuberculosis cycA*-specific primers described above. *M. smegmatis*::pYUB412 served as control. Table 1 describes all strains generated in this study.

**Table 1:** DNA and bacterial strains used in this work.

*Plasmid/Cosmid*	*Description*	*Reference*
**pMD31**	Episomal, multicopy, Kan^R^, *oriE, oriM*	[30]
**pMDcycA_Mtb_**	Episomal, multicopy, *M. tb cycA*, Kan^R^, *oriE, oriM*	This study
**pMDcycA_Mbov_**	Episomal, multicopy, *M. bovis cycA*, Kan^R^, *oriE, oriM*	This study
**pMDcycA_BCG_**	Episomal, multicopy, BCG *cycA*, Kan^R^, *oriE, oriM*	This study
**pYUB412**	*attB* site integrative cosmid backbone, Hyg^R^, Amp^R^, *oriE*	[28]
**I425**	*attB* site integrative cosmid with *rv1701* to *rv1733c* fragment, Hyg^R^, Amp^R^, *oriE*	[28]
***Strains***	***Description***	***Reference***
***M. tb*** ** H37Rv**	Wild-type	[36]
***M. smegmatis*** ** mc^2^155**	Wild-type	[28]
***M. bovis*** ** BCG-Pasteur 1173P2**	Vaccine strain	[1]
**Pasteur::pYUB412**	BCG-Pasteur::pYUB412, Hyg^R^	This study
**Pasteur::I425**	BCG-Pasteur::I425, Hyg^R^	This study
**mc^2^155::pYUB412**	*M. smegmatis* mc^2^155::pYUB412, Hyg^R^	This study
**mc^2^155::I425**	*M. smegmatis* mc^2^155::I425, Hyg^R^	This study
**mc^2^155/pMD31**	*M. smegmatis* mc^2^155+ pMD31, Kan^R^	This study
**mc^2^155/pMDcycA_Mtb_**	*M. smegmatis* mc^2^155+ pMDcycA_Mtb_, Kan^R^	This study
**mc^2^155/pMDcycA_Mbov_**	*M. smegmatis* mc^2^155+ pMDcycA_Mbov_, Kan^R^	This study
**mc^2^155/pMDcycA_BCG_**	*M. smegmatis* mc^2^155+ pMDcycA_BCG_, Kan^R^	This study

### Plasmid Constructions, Generation and Identification of Recombinant *M. smegmatis* Strains Over-expressing *M. tuberculosis*, *M. bovis* and BCG CycA

Multi-copy episomal vectors containing *cycA* alleles from *M. tuberculosis*, *M. bovis* and *M. bovis* BCG were constructed as follows: *cycA* gene including 267 bp upstream of the start site was amplified from *M. tuberculosis* H37Rv, *M. bovis* AF2122/97 and *M. bovis* BCG-Pasteur 1173P2 genomic DNA using high fidelity *Pfu* polymerase and primers *cycA-Hind*III-Forward: cag***aagctt***atcggtgccgcccaact and *cycA-Pst*I-Reverse: cgc***ctgcag***cgtgggcgaggacatag introducing 5′-*Hind*III (bold italics) and 3′-*Pst*I (bold italics) restriction sites. The PCR fragments obtained were digested with *Hind*III and *Pst*I, purified and then ligated into *Hind*III- and *Pst*I- linearized pMD31 [30]. Plasmid clones containing *cycA* alleles were verified by sequencing before electroporation into *M. smegmatis* mc^2^155. Positive transformants were identified by colony PCR using the primers *cycA-*HindIII-Forward and *cycA-*PstI-Reverse described above.

### D-cycloserine Susceptibility Testing

DCS is unstable in aqueous solutions buffered at pH 7 including Middlebrook 7H9 broth [8], [31] therefore working solutions of the antibiotic were prepared freshly in sodium phosphate buffer (pH 9.0) just before use. Two-fold serial dilutions of DCS covering the required range of concentrations were prepared in 7H9 broth (supplemented with ADC and 0.05% Tween-80) for all experiments. For assessing DCS-sensitivity over time, the antibiotic was added to 7H9 broth (supplemented with ADC and 0.05% Tween-80) at approximately 2X the reported MIC per strain for every experiment. Colony forming units at different time points were determined by 7H11 agar plate spreading of serially diluted aliquots of cells.

### Statistics

The unpaired, two-tailed *t* test was used to assess the statistical significance in differences when comparing experimental groups using the GraphPad Prism program (http://www.graphpad.com).

### CycA Amino Acid Sequence Analyses and Helical Trans-membrane Domain Modeling

Alignments of CycA amino acid sequences of different mycobacteria obtained from TubercuList, MarinoList and SmegmaList genome browsers (http://mycobrowser.epfl.ch/) [32] were done using the MultAlin server (http://multalin.toulouse.inra.fr/multalin/multalin.html) [33]. Secondary structure prediction was done using the PSIPRED protein structure prediction server (http://bioinf.cs.ucl.ac.uk/psipred/) [34]. Prediction of trans-membrane domains in CycA amino acid sequences was done using the HMMTOP (version 2) algorithm [21]. Two-dimensional graphical representations of the trans-membrane topologies of CycA sequences were made using the TMRPres2D program (http://bioinformatics.biol.uoa.gr/TMRPres2D/index.jsp) [35].

## Supporting Information

Figure S1 **2-dimensional topological representation of** ***E. coli*** **CycA with a DCS-resistance associated T114P mutation (circled) in the extracellular loop (boxed) between the 3^rd^ and 4^th^ trans-membrane helices from the amino terminus.**
(TIF)
