# Supplementary figures and images for: A Point Mutation in cycA Partially Contributes to the D-cycloserine Resistance Trait of Mycobacterium bovis BCG Vaccine Strains
Source: PLoS One. 2012 Aug 17;7(8):e43467. doi: 10.1371/journal.pone.0043467 (PMC3422274; doi:10.1371/journal.pone.0043467)

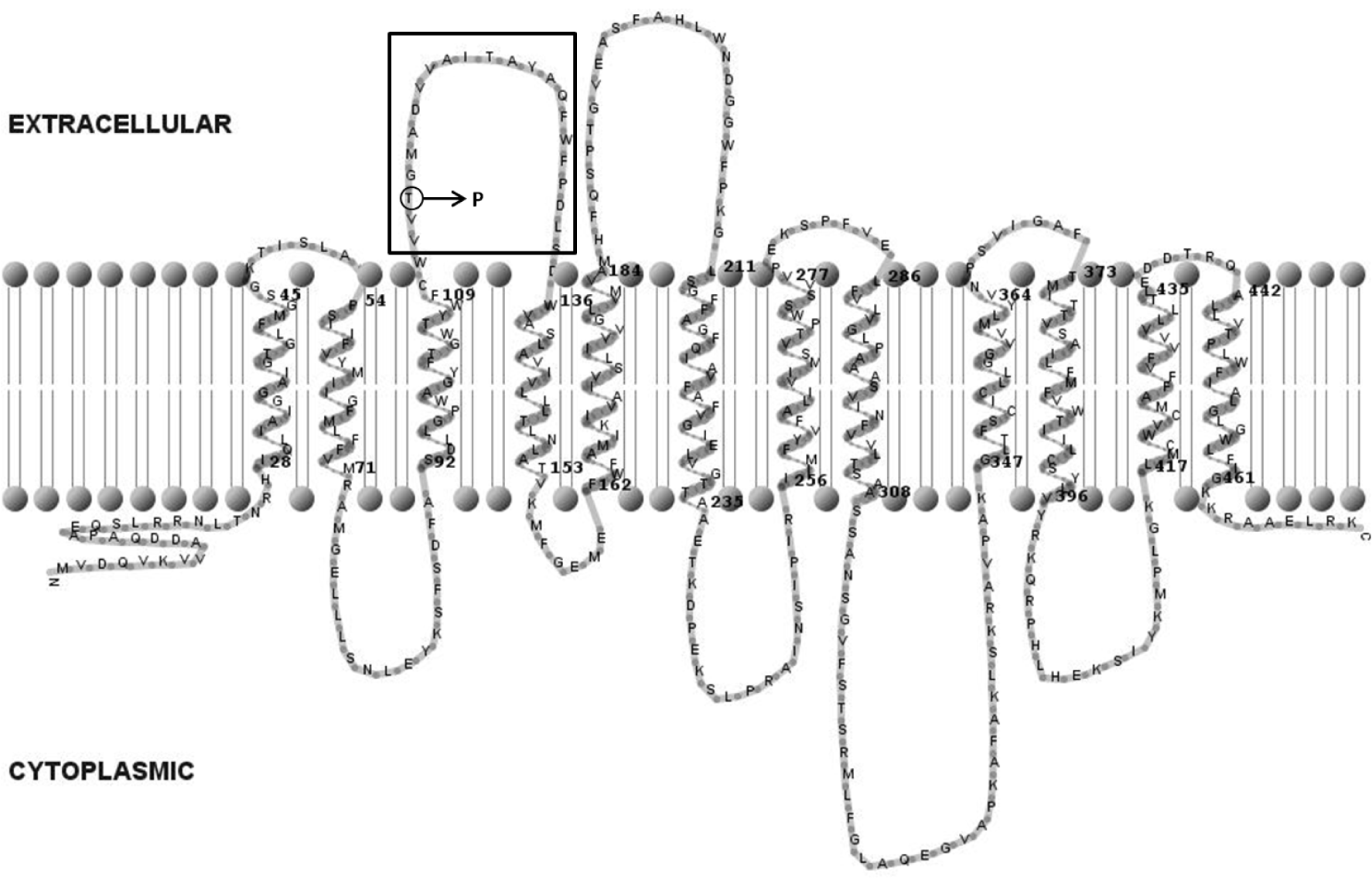

Supplement: Figure S1 — 2-dimensional topological representation of E. coli CycA with a DCS-resistance associated T114P mutation (circled) in the extracellular loop (boxed) between the 3rd and 4th trans-membrane helices from the amino terminus. (TIF) [file pone.0043467.s001.tif]
